# Targeted Sanger sequencing to recover key mutations in SARS-CoV-2 variant genome assemblies produced by next-generation sequencing

**DOI:** 10.1099/mgen.0.000774

**Published:** 2022-03-16

**Authors:** Lavanya Singh, James E. San, Houriiyah Tegally, Pius M. Brzoska, Ugochukwu J. Anyaneji, Eduan Wilkinson, Lindsay Clark, Jennifer Giandhari, Sureshnee Pillay, Richard J. Lessells, Darren Patrick Martin, Manohar Furtado, Anmol M. Kiran, Tulio de Oliveira

**Affiliations:** ^1^​ KwaZulu-Natal Research Innovation and Sequencing Platform, University of KwaZulu-Natal, Durban, South Africa; ^2^​ Thermo Fisher Scientific, South San Francisco, CA, USA; ^3^​ Centre for Epidemic Response and Innovation (CERI), School of Data Science and Computational Thinking, Stellenbosch University, Stellenbosch, 7600, South Africa; ^4^​ HPCBio, Roy J. Carver Biotechnology Center, University of Illinois, IL, USA; ^5^​ Institute of Infectious Diseases and Molecular Medicine, Division of Computational Biology, Department of Integrative Biomedical Sciences, University of Cape Town, Cape Town 7701, South Africa; ^6^​ Malawi-Liverpool-Wellcome Trust, Chichiri, Blantyre 3, Malawi; ^7^​ Institute of Infection, Veterinary and Ecological Sciences, University of Liverpool, Liverpool CH64 7TE, UK; ^8^​ Department of Global Health, University of Washington, Seattle, WA, USA

**Keywords:** Illumina, Sanger, whole-genome sequencing, SARS-CoV-2 spike gene, S-gene, mutations, primer binding site, diagnostic failure

## Abstract

Severe acute respiratory syndrome coronavirus 2 (SARS-CoV-2) is adaptively evolving to ensure its persistence within human hosts. It is therefore necessary to continuously monitor the emergence and prevalence of novel variants that arise. Importantly, some mutations have been associated with both molecular diagnostic failures and reduced or abrogated next-generation sequencing (NGS) read coverage in some genomic regions. Such impacts are particularly problematic when they occur in genomic regions such as those that encode the spike (S) protein, which are crucial for identifying and tracking the prevalence and dissemination dynamics of concerning viral variants. Targeted Sanger sequencing presents a fast and cost-effective means to accurately extend the coverage of whole-genome sequences. We designed a custom set of primers to amplify a 401 bp segment of the receptor-binding domain (RBD) (between positions 22698 and 23098 relative to the Wuhan-Hu-1 reference). We then designed a Sanger sequencing wet-laboratory protocol. We applied the primer set and wet-laboratory protocol to sequence 222 samples that were missing positions with key mutations K417N, E484K, and N501Y due to poor coverage after NGS sequencing. Finally, we developed SeqPatcher, a Python-based computational tool to analyse the trace files yielded by Sanger sequencing to generate consensus sequences, or take preanalysed consensus sequences in fasta format, and merge them with their corresponding whole-genome assemblies. We successfully sequenced 153 samples of 222 (69 %) using Sanger sequencing and confirmed the occurrence of key beta variant mutations (K417N, E484K, N501Y) in the S genes of 142 of 153 (93 %) samples. Additionally, one sample had the Y508F mutation and four samples the S477N. Samples with RT-PCR *C*_t_ scores ranging from 13.85 to 37.47 (mean=25.70) could be Sanger sequenced efficiently. These results show that our method and pipeline can be used to improve the quality of whole-genome assemblies produced using NGS and can be used with any pairs of the most used NGS and Sanger sequencing platforms.

## Impact Statement

Genomic epidemiology has recently come of age as the main tool to inform the introduction, evolution and spread of pathogens. Its dependence on high-quality, whole, or near-whole genomes is, however, affected by the rapid accumulation of mutations in viral pathogens that, among other problems in amplicon-based methods, result in failure to amplify targeted genomic regions when they occur in primer binding sites. In severe acute respiratory syndrome coronavirus 2 (SARS-CoV-2), the emergence of variants of concern has been associated with primer failures, especially in the Alpha/B.1.1.7, Beta/B.1.351, Delta/B.1.672.2 and Omicron/B.1.1.529 variants. This work presents a quick, cost-effective and easy-to-use method and tools to extend coverage in important regions of near-whole genomes. Our method has been applied successfully to fill gaps in the SARS-CoV-2 receptor binding domain of the Beta variant and can be extended to any gene and to other pathogens, significantly improving the quality of genomic data available to inform public health decisions. Most importantly, we provide an end-to-end solution incorporating both the wet-laboratory and bioinformatics components that can be easily adapted by other laboratories to enhance the utility of already sequenced near-whole genomes.

## Data Summary

The Sanger sequencing protocol can be accessed at: https://www.protocols.io/view/targeted-sequencing-by-sanger-to-recover-key-mutat-buhint4e.

The SeqPatcher tool is also freely available at: https://github.com/krisp-kwazulu-natal/seqPatcher. All other data have been provided within the article or in the supplementary files including GISAID IDs for the sequences analysed in this study. Raw sequence data have been deposited in the National Center for Biotechnology Information (NCBI) Short Read Archive (SRA) under the project number PRJNA636748.

## Introduction

Severe acute respiratory syndrome coronavirus 2 (SARS-CoV-2) genomes of high quality and coverage are increasingly needed to support public health efforts to bring the coronavirus disease 2019 (COVID-19) pandemic to an end [1]. These data are needed to detect the emergence of variants of concern (VOCs) and variants of interest (VOIs) and track their prevalence and spread around the world. Of particular interest is the spike (S) gene, which is both a major determinant of viral infectivity [2, 3] and the primary target of virus-induced neutralizing antibodies (Nabs) [4] and, as such, is the site of multiple known mutations that enhance viral transmission and/or enable viral escape from the immune system [5]. As a result of this, the S gene exhibits markedly higher evolution rates than other genes in the SARS-CoV-2 genome [6, 7]. This makes the S gene an important target for viral diversity, transmission, pathogenicity and evolution studies. In addition to the potential immunological and epidemiological implications of mutations in the S gene, such mutations can also affect the binding, and therefore the utility, of the primers and probes that are used to diagnose and sequence SARS-CoV-2 [8]. Continuous assessments of the occurrence and spread of mutations in the S gene are therefore a crucial component of efforts to manage the pandemic.

Short-read sequencing technologies such as Illumina can enable the scale and speed of whole-genome sequencing that is required for successful near real-time SARS-CoV-2 genomic surveillance [9]. However, the quality and depth of sequence data that are obtained with short-read sequencing platforms for any given genome can vary considerably between different regions of the genome [10, 11]. For example, the S gene codon 69–70 deletion in the Alpha/B.1.1.7 lineage has been noted to cause spike gene target failures (SGTFs) in diagnostic tests [12, 13]. The presence of mutations, especially minority variants, can also affect the annealing of primer sequences and reduce the efficiency of sequencing [14].

Several options to augment read coverage at target genome loci have been explored, including increasing the amounts of DNA that are used for next-generation sequencing (NGS), the use of DNA capture agents, and optimized DNA purification and amplification protocols, such as those employing DNA concentration steps or the sequencing of smaller amplified fragments [11]. Although effective in many instances, in others the quality and not the quantity of the available DNA is the limiting factor. Post-sequencing computational tools such as GapFiller [15] have also been developed in an attempt to extend the quantity of readable sequence that can be obtained from short reads *in silico*; however, the high confidence in basecalling needed for genomic surveillance limits the use of imputation-based methods. Altogether, these efforts highlight the extent of a persistent issue with the rapidly accumulating pool of genomic surveillance data; that is, the pervasiveness of significant gaps in near whole-genome sequences that are being deposited in public sequence repositories.

Targeted gene-specific Sanger sequencing is a speedy, cost-effective and accurate means of sequencing genomic regions to fill in small gaps in partial whole genomes determined using NGS [11]. Specifically, short fragments (<1000 bp) from particular genome regions of interest, for example the region of the S gene encoding the receptor binding domain (RBD), can be amplified, sequenced and used to ‘patch’ near full-genome assemblies to produce higher quality genome sequences. This is especially useful when regions of low read coverage correspond to key sites of the genome known to harbour mutations of interest, such as the spike E484K and N501Y, which are mutations associated with escape of neutralizing and monoclonal antibodies, respectively [16, 17], and the K417N/T, which has been implicated in increased transmissibility due to elevated angiotensin converting enzyme - 2 (ACE2) receptor binding affinity [18].

Here we describe a targeted Sanger sequencing approach that can be used to sequence sub-regions of any gene to fill in the gaps remaining following whole-genome sequencing by NGS. We demonstrate the utility of the approach by using it to restore sequencing coverage in the RBD region of the S gene of the Beta (501Y.V2/B.1.351) lineage viruses that harbour the signature spike mutations K417N, E484K and N501Y. Specifically, we developed a set of custom primers, a Sanger sequencing protocol and a computational tool to recover mutations occurring in low/missing-coverage regions of whole-genome sequences. We show that we were able to detect the presence of the K417N, E484K and N501Y mutations accurately in 142 samples with *C*_t_ scores between 13.85–37.47. Our results demonstrate that Sanger sequencing data can be easily integrated with NGS-derived whole-genome sequencing data or used on their own for rapid genotyping.

## Methodology

### Data

The samples analysed in this study include 222 SARS-CoV-2 nasopharyngeal swab samples from routine surveillance during the second wave of the pandemic in South Africa. Sample collection dates ranged between December 2020 and January 2021 at the peak of the epidemic dominated by the beta variant. All samples analysed were missing coverage in the RBD region of the genome after sequencing with NGS on the Illumina platform.

### PCR primers

We designed sequencing primers to target the 401 base pair (bp) region between genome coordinates 22 698 and 23 098 of the SARS-CoV-2 Wuhan-Hu-1 reference (NC_045512.2) genome, which included mutation sites K417N (G22813T), E484K (G23012A) and N501Y (A23063T) of the beta variant of concern (VOC). The primers were designed using primer3 v4.1.3 [19–21] following Brzoska *et. al* [22].

### cDNA synthesis and PCR

Briefly, RNA was extracted from samples using standard techniques; 10 µl of RNA (1 pg–100 ng) was used in a total reaction volume of 20 µl, using the one-step cDNA and PCR protocol (TaqPath 1-step RT-qPCR MasterMix, Thermo Fisher Scientific) and 501Y.V2 forward and reverse primers (fwd: GATCTCTGCTTTACTAATGTCTATGCAGAT, rev: GCTGGTGCATGTAGAAGTTCAAAAG). PCR reactions underwent thermocycling at the following conditions: Uracil-N-glycosylase (UNG) incubation at 25 °C for 2 min, reverse transcriptase (RT) incubation at 50 °C for 15 min and enzyme activation at 95 °C for 2 min, followed by 40 cycles of amplification at 95 °C for 3 s and 60 °C for 30 s.

### Cycle sequencing and capillary electrophoresis

Ten microlitres of PCR amplicon was purified (ExoSap IT-express reagent, Thermo Fisher Scientific) and 1 µl of product used for cycle sequencing (BigDye Terminator v3.1 cycle sequencing kit, Thermo Fisher Scientific) using the same 501Y.V2 forward and reverse primers that were used in the amplification step. The products were then purified (BigDye Xterminator kit, Thermo Fisher Scientific) and capillary electrophoresed using a DNA Analyser (Applied Biosystems). The detailed step-by-step wet-laboratory protocol is available on protocols.io at: https://www.protocols.io/view/targeted-sequencing-by-sanger-to-recover-key-mutat-buhint4e.

### Bioinformatics analysis and algorithm

Following capillary electrophoresis, the resulting sequence traces were visually inspected in Geneious Prime. The traces were then analysed and merged into the whole genome sequences using our custom tool, SeqPatcher. The tool accepts as input either trace (.ab1) files produced by the Sanger sequencing device or the consensus sequences from preanalysed trace files, a reference sequence of the gene of interest and one or more near-complete genome(s) with missing data, usually represented as Ns in the region of interest. The tool assumes that the gene of interest has only been partially sequenced in the near-complete genome(s), such that the location of the gene of interest can be determined by alignment.

If trace files are provided, SeqPatcher checks that the length is greater than 100 bp and computes the median peak height of the bases within the interquartile range of the sequence. A warning is issued for sequences of length lower than 100 bp. Low-quality bases at the ends of the sequence with peaks <10 % of the median height are trimmed.

**Fig. 1.**
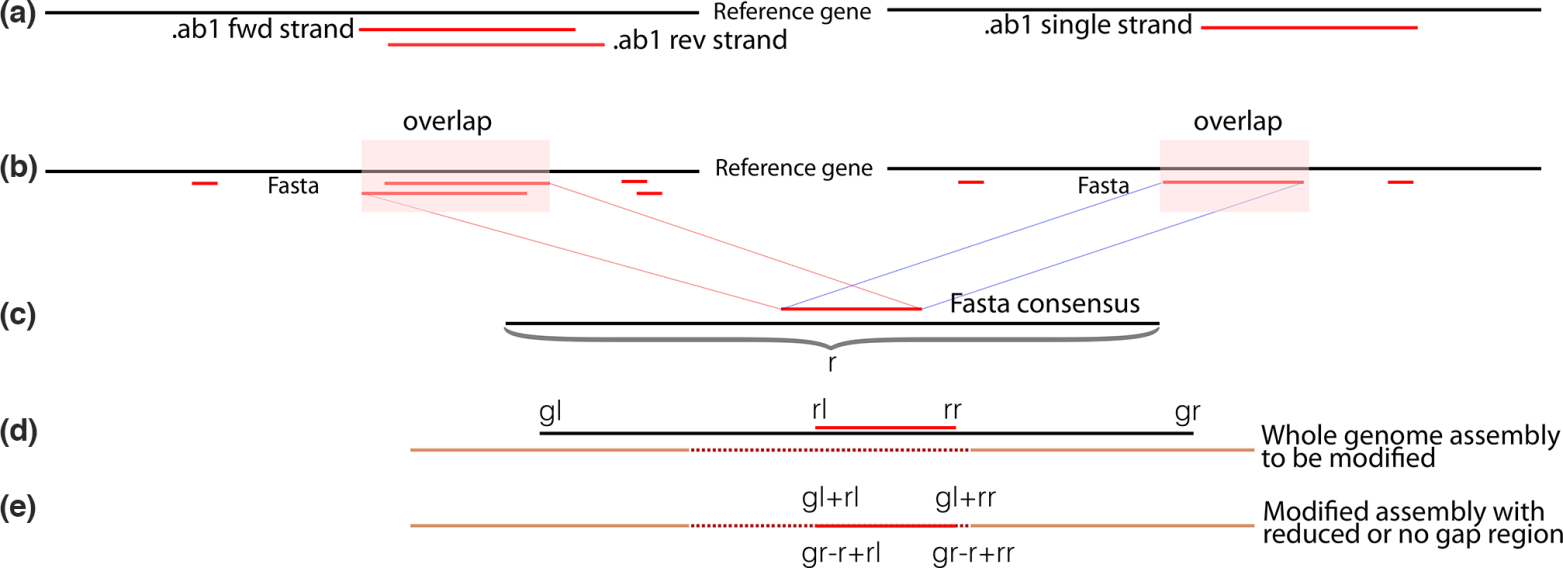
Visual representation of the SeqPatcher workflow. (**a**) If the input is an .ab1 file then SeqPatcher analyses and infers consensus sequences in fasta format. (**b**) fasta sequences are aligned to the reference gene and (c) the longest overlap is determined. The location of the overlap relative to the gene is also determined. (**d**) The reference gene is then aligned to the respective whole genome to determine the location and the precise point of insertion is calculated. (**e**) Sanger sequence is inserted into the whole genome. SeqPatcher tries to report the correct/highly probable base in cases of conflict and keeps the locations of indels (insertions and deletions) intact during integration. r, ref gene length; rl, ref gene start position; rr, ref gene end position; gl, gene start position in assembly; gr, gene end position in assembly; black solid line, reference gene; dots, missing or gapped region in the sequence.

The tool then analyses the remaining peaks to determine the prevalent base at each position and produces a fasta sequence. The sequence is pairwise aligned with the reference gene using muscle [23] to determine the longest contiguous aligned region (Fig. 1a, b). Where paired reads are provided (forward and reverse), the tool calculates a consensus. Mismatches between the overlapping region of the forward and reverse reads as well as ambiguous bases are resolved as shown in Table 1. InDels of length >15 nt or that are not a multiples of three in the Sanger sequences are included or excluded (i.e. insertions removed and deletions replaced with Ns) based on user provided input. The coordinates of the region to be replaced are determined relative to the reference gene. The region matching the reference gene within the whole-genome assembly is then ascertained using blat [24]. The precise location to be modified is calculated as shown in Fig. 1d, e, and the Sanger sequence is inserted into the whole-genome sequence. The final output is a file containing all the genomes. Only genomes that have a corresponding Sanger sequence are edited, relieving the user of the need to manually organize and process files in situations involving large numbers of sequences. The consensus Sanger sequences in fasta format are also output for the user. Additional detail of the algorithm has been provided in the documentation (https://github.com/krisp-kwazulu-natal/seqPatcher).

**Table 1.** Derivation of the Sanger consensus sequence from the trace files. A fasta sequence is determined for each read and a consensus calculated by alignment of the reads to the reference gene sequence. Base refers to nucleotides A, C, T and G and A/B refer to different bases at that position. A dash (–) represents an indel

Sanger sequence with forward and reverse reads			
Reference	Forward read	Reverse read	Final outcome
–	Any base	Any base	Any base
Base A	Base A	Base A	Based on user input, i.e. based on peak heights or ambiguous base (default: ambiguous base)
Base A	Base A	Base B	Based on user input, i.e. based on peak heights or ambiguous base (default: ambiguous base)
Base A	Base B	Base A	Base A
Base A	Base B	Base B	Base B
Base A	Base B	–	Base B
Base A	Base A	–	Base A
Base A	Ambiguous	Base B	Base B
Base A	Base B	Ambiguous	Base B
Sanger sequence with only one read (either forward or reverse)			
–	Any base		Any base
Base A	–	na	–
Base A	Base B	na	Base B
Base A	Ambiguous	na	Based on user input, base a/ peak max base/ neighbour base/ base ambi. (Default: base ambi.)

Only the near-complete genome sequences with a matching Sanger sequence are merged by SeqPatcher, such that the user does not need to first separate the sequences that need to be merged from an existing dataset of complete and near-complete sequences.

### Validation

We analysed 153 sets of trace files from samples that were successfully Sanger sequenced. The trace files were analysed in both Geneious Prime and SeqPatcher to generate consensus sequences. The resulting consensus sequences were analysed in Nextclade (https://clades.nextstrain.org/) and the mutational sets were compared. Nextclade produces a report detailing sequence quality and coverage and identifies mutational changes observed at the nucleotide and protein levels relative to the Wuhan reference.

To assess the merging step, we generated a Nextclade report of the whole-genome sequence before merging to determine the detectable mutations present at baseline. Next, we generated a Nextclade report of the Sanger sequences to identify the mutations recovered within these. Finally, we generated a Nextclade report of the mutations present in the consensus genomes after merging.

The Nextclade report of the Sanger sequences enabled us to identify instances where merging the Sanger and NGS data yielded a composite genome sequence assembly within which the expected mutation profiles were and were not successfully detected, while the report after merging was used to confirm a successful merge.

## Results

### Sanger sequencing

**Fig. 2.**
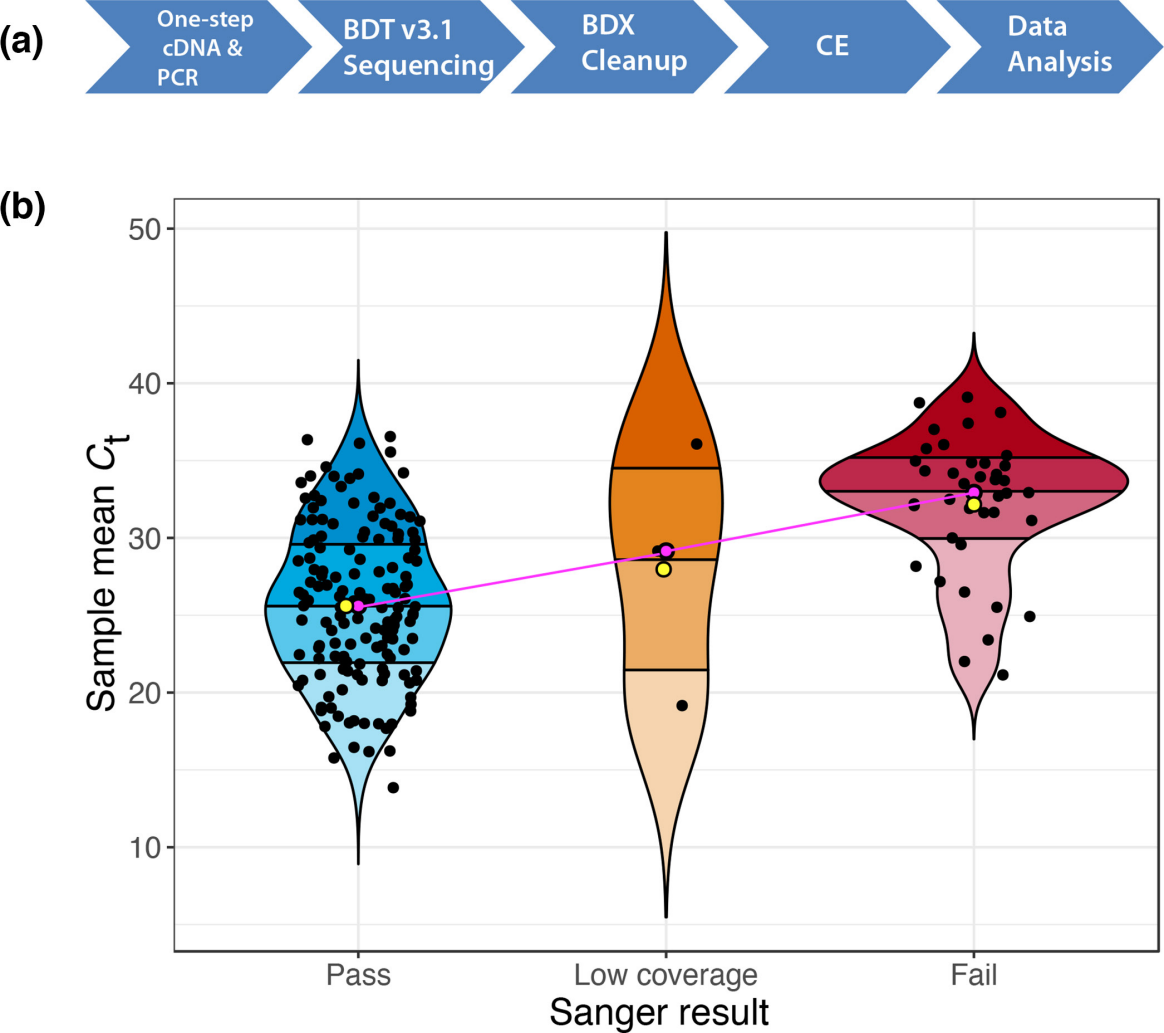
Sanger sequencing and analysis. (**a**) The Sanger sequencing workflow. cDNA, complementary DNA; PCR, polymerase chain reaction; BDT, BigDye Terminator; BDX, BigDye Xterminator; CE, capillary electrophoresis. (**b**) Violin plot showing the distribution of RT-PCR cycle threshold scores for 222 samples; the *C*_t_ score for each sample was based on the mean *C*_t_ of the three SARS-CoV-2 targets (S gene, N gene, Orf1ab). Quartiles are represented by different shades within each plot, mean and median *C*_t_s are represented by yellow and pink points, respectively. The pink line shows the trend line.

We performed Sanger sequencing on 222 samples that, following sequencing with the Illumina platform, had yielded near-complete genome sequences that lacked part of the S gene (Table 1). Of these, 153 samples with a mean *C*_t_ 25.70 (range: 13.85–37.47) were successfully Sanger sequenced, yielding at least 1 complete sequence (forward or reverse) that spans the region of interest (between 22 698 and 23 098, i.e. 401 bp relative to the Wuhan-Hu-1 reference). Five samples, mean *C*_t_ 29.26 (19.16–36.07), yielded poor quality Sanger sequence with insufficient coverage of the region of interest and were therefore discarded, while 69 samples with a mean *C*_t_ of 31.81 (20.78–38.92) did not yield a Sanger sequence and were therefore not analysed further. Fig. 2a shows our optimized Sanger sequencing protocol and Fig. 2b shows a summary of the sequencing results. Additional mutations (Y508F and S477N) associated with the S gene were also detected in five of the samples (Table S1).

### Sequence merging

Using SeqPatcher, we inferred a consensus sequence for all samples that were successfully Sanger sequenced. The consensus sequences were successfully merged into the whole-genome sequences for all 153 of the cases. The success of the merge process was confirmed using Nextclade reports. All sequences with genome coverage >80% were deposited on GISAID (Table S2). Table S3 shows a Nextclade report for all the samples submitted to GISAID.

**Fig. 3.**
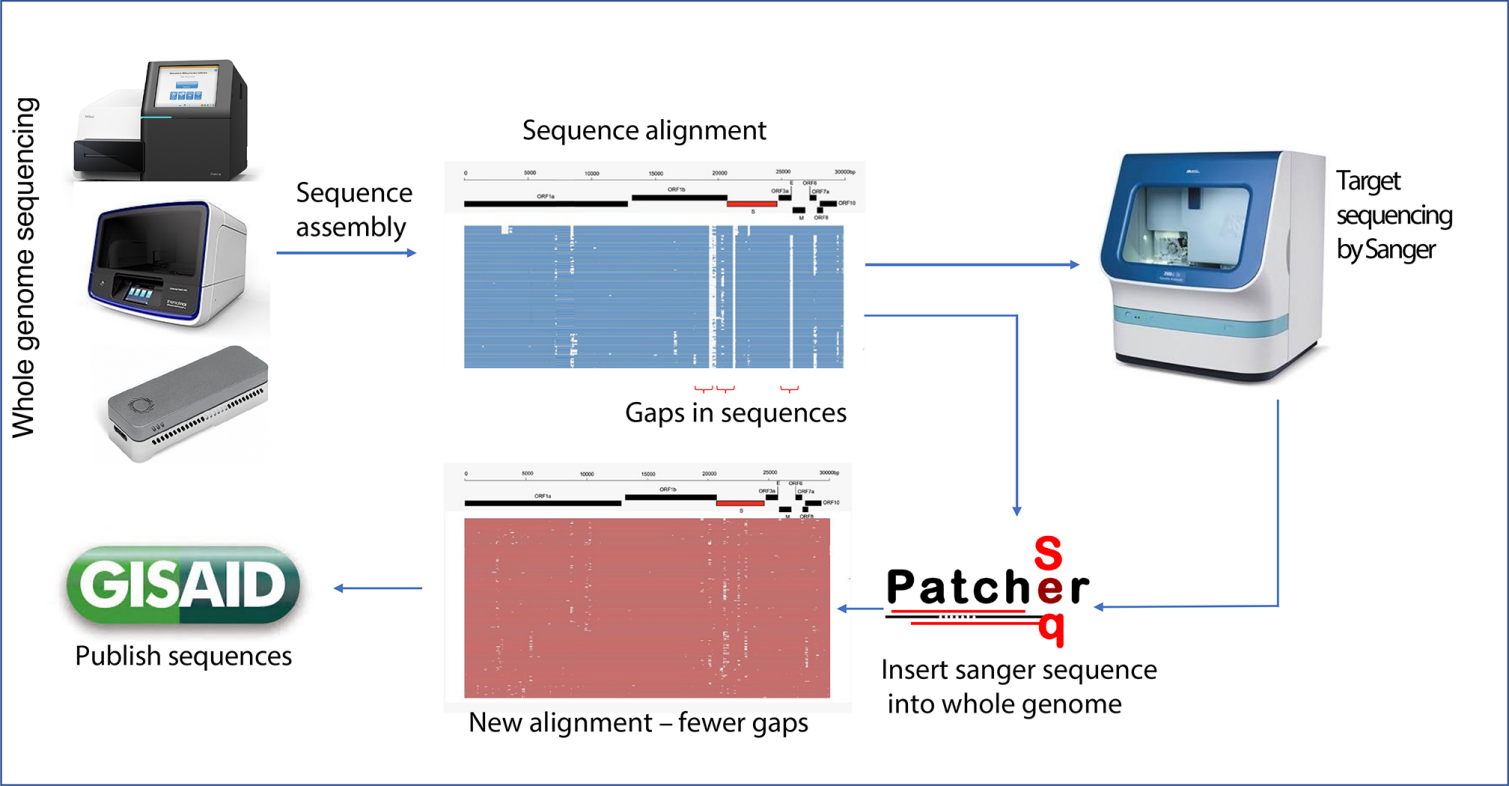
Summary of the workflow for improving the coverage of WGS data using Sanger sequencing. Data from the sequencer are preprocessed to determine coverage. Sequences with gaps in regions of interest are sent for Sanger sequencing. The results from Sanger sequencing are inserted into the NGS whole genomes using SeqPatcher and the improved consensus of these is published to support genomic epidemiology.

A graphical summary of the workflow to incorporate Sanger sequences into the near-complete NGS-generated genome sequences is presented in Fig. 3.

### Future directions

Future development directions include the implementation of a graphical interface for this analysis and for viewing the merged sequences for users that are unfamiliar with using command-line tools.

## Discussion

Sanger sequencing has been suggested to be the most useful technique to sequence short fragments needed to patch near-complete genome assemblies following the failure of diagnostic assays [25], a common phenomenon when these assays target rapidly evolving regions of the genome. In the current study, we incorporated Sanger sequencing using a custom primer set that targets the RBD of the S gene of SARS-CoV-2, a region that commonly has low read coverage during NGS. The successful application of our method demonstrates the utility of Sanger sequencing in improving the coverage and quality of whole genomes derived from short-read sequencing.

We performed RT-PCR on the samples to investigate the upper bounds of cycle threshold (*C*_t_) values at which Sanger sequencing would be viable. While our results show that *C*_t_ values below 25 are suitable for the reliable application of Sanger sequencing, we managed to successfully Sanger sequence samples with *C*_t_ values as high as 37.5. The Sanger sequencing RT-PCR-based protocol described here enabled sequence data to be generated both rapidly and reliably. Specifically, there is a one-step cDNA synthesis and PCR workflow that can be completed in approximately 6 h, which compares very favourably with the >30 h of sample preparation required for short-read sequencing technologies such as Illumina.

Sixty-nine of the 222 samples failed Sanger sequencing; this was either due to failure to amplify by PCR and/or the cycle sequencing reaction(s) or because the resultant consensus sequences contained gaps (due to poor primer coverage). While most of these samples typically had high *C*_t_ scores, others were well within the *C*_t_ range of those that could be successfully sequenced (mean *C*_t_=25.70). The failure of these samples could possibly be attributed to poor sample quality or RNA degradation (due to multiple freeze–thaw events, for example). Key mutations were not found in 11 out of the 153 samples that had been successfully Sanger sequenced.

A major challenge with analysing raw Sanger sequence data is the high cost associated with tools such as Geneious and Thermo Fisher’s proprietary sequencing analysis software, SeqA. Additionally, software such as SeqA is computing platform-dependent, which seriously limits its broad utility. SeqPatcher is free and utilizes dependences that are freely available or with functionality that is available entirely as open source. The codebase is implemented in Python, a platform-independent language, implying that it is accessible regardless of the user’s computing platform. Users are free to use as well as modify SeqPatcher to suit their personal needs. Our pipeline also adheres to key principles of scientific software development, such as ‘run as a single command’ and ‘extensive documentation’ to ensure there is no technical barrier to usage [26]. Our method thus encourages a do-it-yourself analytical framework.

While SeqPatcher was developed to facilitate the completion of SARS-CoV-2 whole-genome sequences, the software enables the merging of any two related sequences. SeqPatcher also extends the utility of existing sequence analysis tools such as Biopython Bio.SeqIO [27], which provide functionality to analyse .abi formatted trace files through its specialized functions to integrate the Sanger sequence fragments into their corresponding whole genomes.

Various tools such as GapFiller [15] have been developed in the past to extend the coverage of short-read whole-genome sequence assemblies *in silico*. These tools were mostly developed on the assumption of poor assembly, often as a result of complex genomic structural features, such as tandem repeats. However, assembly methods have improved significantly. Advanced assemblers such as metaSpades [28] and megahit [29] are capable of powerful methodologies, including *de novo*, reference guided and graph methodologies. Assembly applications such as GenomeDetective [30] combine the power of these assemblers with alignment and mapping tools such as aga [31], muscle [23] and Minimap2 [32] to ensure that the most optimal and accurate assembly of sequenced reads is obtained. As such, in many instances, gapped regions in a sequence represent true failure of diagnostics to amplify a targeted region due to primer mismatch, for example, and not the misplacement of reads. On the other hand, the sensitive nature of genomic surveillance requires very high confidence in called mutations, leaving little room for imputation. Employing a hybrid approach combining targeted sequencing with bioinformatic tools thus provides an accurate, feasible and cost/time-effective solution that can be integrated into any sequence generating laboratory to quickly increase the rates at which high-quality whole-genome sequences can be produced.

## Conclusion

Targeted Sanger sequencing of short sequence fragments has the potential to increase the number and quality of whole genomes produced in the pursuit of global pathogen genomic surveillance. In the case of rapidly evolving pandemics caused by pathogens such as SARS-CoV-2 and HIV, routine updates of workflows and primer sets will be essential to ensure that degrees of sequencing coverage and quality do not begin to decline as pandemics progress.

## Supplementary Data

Supplementary material 1

## References

[1] Li Q, Wu J, Nie J, Zhang L, Hao H (2020). The impact of mutations in SARS-CoV-2 spike on viral infectivity and antigenicity. Cell.

[2] Korber B, Fischer WM, Gnanakaran S, Yoon H, Theiler J (2020). Tracking changes in SARS-CoV-2 spike: evidence that d614g increases infectivity of the COVID-19 virus. Cell.

[3] Zhang L, Jackson CB, Mou H, Ojha A, Peng H (2020). SARS-CoV-2 spike-protein D614G mutation increases virion spike density and infectivity. Nat Commun.

[4] He Y, Zhou Y, Liu S, Kou Z, Li W (2004). Receptor-binding domain of SARS-CoV spike protein induces highly potent neutralizing antibodies: implication for developing subunit vaccine. Biochem Biophys Res Commun.

[5] VanBlargan LA, Goo L, Pierson TC (2016). Deconstructing the antiviral neutralizing-antibody response: implications for vaccine development and immunity. Microbiol Mol Biol Rev.

[6] Yin C (2020). Genotyping coronavirus SARS-CoV-2: methods and implications. Genomics.

[7] Tang X, Wu C, Li X, Song Y, Yao X (2020). On the origin and continuing evolution of SARS-CoV-2. Natl Sci Rev.

[8] Cotten M, Lule Bugembe D, Kaleebu P, V T Phan M (2021). Alternate primers for whole-genome SARS-CoV-2 sequencing. Virus Evol.

[9] Illumina (2021). Key differences between next-generation sequencing and Sanger sequencing: Understanding when NGS can be a more effective option. https://emea.illumina.com/science/technology/next-generation-sequencing/ngs-vs-sanger-sequencing.html.

[10] Nasir JA, Kozak RA, Aftanas P, Raphenya AR, Smith KM (2020). A comparison of whole genome sequencing of SARS-CoV-2 using amplicon-based sequencing, random hexamers, and bait capture. Viruses.

[11] Hagemann IS (2015). Clinical Genomics.

[12] Borges V, Sousa C, Menezes L, Gonçalves AM, Picão M (2021). Tracking SARS-CoV-2 lineage B.1.1.7 dissemination: insights from nationwide spike gene target failure (SGTF) and spike gene late detection (SGTL) data, Portugal, week 49 2020 to week 3 2021. Euro Surveill.

[13] Bal A, Destras G, Gaymard A, Stefic K, Marlet J (2021). Two-step strategy for the identification of SARS-CoV-2 variant of concern 202012/01 and other variants with spike deletion H69–V70, France, August to December 2020. Euro Surveill.

[14] Sapoval N, Mahmoud M, Jochum MD, Liu Y, Elworth RAL (2021). SARS-CoV-2 genomic diversity and the implications for qRT-PCR diagnostics and transmission. Genome Res.

[15] Nadalin F, Vezzi F, Policriti A (2012). GapFiller: a de novo assembly approach to fill the gap within paired reads. BMC Bioinformatics.

[16] Greaney AJ, Loes AN, Crawford KH, Starr TN, Malone KD (2021). Comprehensive mapping of mutations in the sars-cov-2 receptor-binding domain that affect recognition by polyclonal human plasma antibodies. Cell Host & Microbe.

[17] Wang Z, Schmidt F, Weisblum Y, Muecksch F, Barnes CO (2021). mRNA vaccine-elicited antibodies to SARS-CoV-2 and circulating variants. Nature.

[18] Cheng MH, Krieger JM, Kaynak B, Arditi M, Bahar I (2021). Impact of south african 501.v2 variant on SARS-CoV-2 spike infectivity and neutralization: a structure-based computational assessment. BioRxiv.

[19] Koressaar T, Remm M (2007). Enhancements and modifications of primer design program Primer3. Bioinformatics.

[20] Untergasser A, Cutcutache I, Koressaar T, Ye J, Faircloth BC (2012). Primer3--new capabilities and interfaces. Nucleic Acids Res.

[21] Kõressaar T, Lepamets M, Kaplinski L, Raime K, Andreson R (2018). Primer3_masker: integrating masking of template sequence with primer design software. Bioinformatics.

[22] Brzoska PM, Brown C, Cassel M, Ceccardi T, Di Francisco V (2006). An efficient and high-throughput approach for experimental validation of novel human gene predictions. Genomics.

[23] Edgar RC (2004). MUSCLE: a multiple sequence alignment method with reduced time and space complexity. BMC Bioinformatics.

[24] Kent WJ (2002). BLAT--the BLAST-like alignment tool. Genome Res.

[25] World Health Organization (2021). Genomic sequencing of SARS-CoV-2: a guide to implementation for maximum impact on public health.

[26] Ewels PA, Peltzer A, Fillinger S, Patel H, Alneberg J (2020). The nf-core framework for community-curated bioinformatics pipelines. Nat Biotechnol.

[27] Cock PJA, Antao T, Chang JT, Chapman BA, Cox CJ (2009). Biopython: freely available Python tools for computational molecular biology and bioinformatics. Bioinformatics.

[28] Nurk S, Meleshko D, Korobeynikov A, Pevzner PA (2017). metaSPAdes: a new versatile metagenomic assembler. Genome Res.

[29] Li D, Liu C-M, Luo R, Sadakane K, Lam T-W (2015). MEGAHIT: an ultra-fast single-node solution for large and complex metagenomics assembly via succinct de Bruijn graph. Bioinformatics.

[30] Cleemput S, Dumon W, Fonseca V, Abdool Karim W, Giovanetti M (2020). Genome Detective Coronavirus Typing Tool for rapid identification and characterization of novel coronavirus genomes. Bioinformatics.

[31] Deforche K (2017). An alignment method for nucleic acid sequences against annotated genomes. BioRxiv.

[32] Li H (2018). Minimap2: pairwise alignment for nucleotide sequences. Bioinformatics.

